# Janowski type close-to-convex functions associated with conic regions

**DOI:** 10.1186/s13660-017-1535-4

**Published:** 2017-10-17

**Authors:** Shahid Mahmood, Muhammad Arif, Sarfraz Nawaz Malik

**Affiliations:** 1 Department of Mechanical Engineering, Sarhad University of Science & I. T, Landi Akhun Ahmad, Hayatabad Link. Ring Road, Peshawar, Pakistan; 2 Department of Mathematics, Abdul Wali Khan University Mardan, Mardan, Pakistan; 3 Department of Mathematics, COMSATS Institute of Information Technology, Wah Cantt, Pakistan

**Keywords:** 30C45, 30C50, close-to-convex functions, Janowski functions, conic domain

## Abstract

The analytic functions, mapping the open unit disk onto petal and oval type regions, introduced by Noor and Malik (Comput. Math. Appl. 62:2209-2217, 2011), are considered to define and study their associated close-to-convex functions. This work includes certain geometric properties like sufficiency criteria, coefficient estimates, arc length, the growth rate of coefficients of Taylor series, integral preserving properties of these functions.

## Introduction and definitions

Let $\mathcal{A}$ be the class of functions *f* of the form

1.1 $$ f ( z ) =z+\sum_{n=2}^{\infty}a_{n}z^{n}, $$

which are analytic in the open unit disk $E= \{ z\in\mathbb{C} : \vert z\vert <1 \} $. Furthermore, $\mathcal{S}$ represents the class of all functions in $\mathcal{A}$ which are univalent in *E*.

The convolution (Hadamard product) of functions $f,g\in\mathcal{A} $ is defined by

$$ (f\ast g) ( z ) =z+\sum_{n=2}^{\infty}a_{n}b_{n}z^{n},\quad z\in E, $$

where $f(z)$ is given by (1.1) and

$$ g(z)=z+\sum_{n=2}^{\infty}b_{n}z^{n},\quad z\in E. $$

For two functions *f* and *g* analytic in *E*, we say that *f* is subordinate to *g*, denoted by $f\prec g$, if there exists a Schwarz function *w* with $w ( 0 ) =0$ and $\vert w ( z ) \vert <1$ such that $f ( z ) =g ( w ( z ) ) $. In particular, if *g* is univalent in *E*, then $f ( 0 ) =g ( 0 ) $ and $f ( E ) \subset g ( E ) $. For more details, see [2].

A function *p* analytic in *E* and of the form

$$ p ( z ) =1+\sum_{n=1}^{\infty}p_{n}z^{n} $$

belongs to the class $\mathcal{P}[A,B]$ if and only if

$$ p ( z ) \prec\frac{1+Az}{1+Bz}, \quad -1\leq B< A\leq1. $$

This class was introduced and investigated by Janowski [3]. In particular, if $A=1$ and $B=-1$, we obtain the class $\mathcal{P}$ of functions with a positive real part (see [4, 5]). The classes $\mathcal{P}$ and $\mathcal{P}[A,B]$ are connected by the relation

$$ p ( z ) \in\mathcal{P}\Leftrightarrow\frac{ ( A+1 ) p ( z ) - ( A-1 ) }{ ( B+1 ) p ( z ) - ( B-1 ) }\in\mathcal{P}[A,B]. $$

Now consider, for $k\geq0$, the classes $k-\mathcal{CV}$ and $k-\mathcal{ST}$ of *k*-uniformly convex functions and corresponding *k*-starlike functions, respectively, introduced by Kanas and Wisniowska, respectively. For some details, see [6–8].

Consider the domain

1.2 $$ \Omega_{k}= \bigl\{ u+iv;u>k\sqrt{ ( u-1 ) ^{2}+v^{2}} \bigr\} . $$

For fixed *k*, $\Omega_{k}$ represents the conic region bounded successively by the imaginary axis ($k=0$), the right branch of a hyperbola ($0< k<1$), a parabola ($k=1$) and an ellipse ($k>1$). This domain was studied by Kanas [6–8]. The function $p_{k}$, with $p_{k} ( 0 ) =1$, $p_{k}^{\prime} ( 0 ) >0$ plays the role of extremal and is given by

1.3 $$ p_{k} ( z ) = \textstyle\begin{cases} \frac{1+z}{1-z}, &k=0, \\ 1+\frac{2}{\pi^{2}} ( \log\frac{1+\sqrt{z}}{1-\sqrt{z}} ) ^{2}, &k=1, \\ 1+\frac{2}{1-k^{2}}\sinh^{2} [ ( \frac{2}{\pi}\arccos k ) \arctan h\sqrt{z} ] ,& 0< k< 1, \\ 1+\frac{1}{k^{2}-1}\sin [ \frac{\pi}{2R ( t ) }\int _{0}^{\frac{u ( z ) }{\sqrt{t}}}\frac{1}{\sqrt{1-x ^{2}}\sqrt{1- ( tx ) ^{2}}}\,dx ] +\frac{1}{k^{2}-1}, &k>1, \end{cases} $$

where $u(z)=\frac{z-\sqrt{t}}{1-\sqrt{tz}}$, $t\in(0,1)$, $z\in E$ and *t* is chosen such that $k=\cos h ( \frac{\pi R^{\prime}(t)}{4R(t)} ) $, with $R(t)$ is Legendre’s complete elliptic integral of the first kind and $R^{\prime}(t)$ is the complementary integral of $R(t)$ (see [7–9]). Let $\mathcal{P} _{p_{k}}$ denote the class of all those functions $p ( z ) $ which are analytic in *E* with $p ( 0 ) =1$ and $p ( z ) \prec p_{k} ( z ) $ for $z\in E$. Clearly, it can be seen that $\mathcal{P}_{p_{k}}\subset\mathcal{P}$, where $\mathcal{P}$ is the class of functions with a positive real part (see [4, 5]). For the applications and exclusive study of the class $\mathcal{P}$, we refer to [10–14]. More precisely

$$ \mathcal{P}_{p_{k}}\subset\mathcal{P} \biggl( \frac{k}{1+k} \biggr) \subset\mathcal{P}, $$

and, for $p\in\mathcal{P}_{p_{k}}$, we have

$$ \bigl\vert \arg p ( z ) \bigr\vert \leq\frac{\lambda \pi}{2}, $$

where

1.4 $$ \lambda=\frac{2}{\pi}\arctan \biggl( \frac{1}{k} \biggr) . $$

Therefore, we can write

$$ p ( z ) =h^{\lambda} ( z ) ,\quad h\in\mathcal{P}. $$

### Definition 1.1

([1])

A function *p* analytic in *E* belongs to the class $k- \mathcal{P} [ A,B ] $ if and only if

$$ p ( z ) \prec\frac{ ( A+1 ) p_{k}(z)- ( A-1 ) }{ ( B+1 ) p_{k}(z)- ( B-1 ) }, \quad k\geq0, $$

where $p_{k}(z)$ is defined by (1.3) and $-1 \leq B< A\leq1$. Geometrically, the function $p ( z ) \in k- \mathcal{P} [ A,B ] $ takes all values in the domain $\Omega_{k} [ A,B ] $, $-1\leq B< A\leq1$, $k\geq0$, which is defined as follows:

$$ \Omega_{k} [ A,B ] = \biggl\{ w:\operatorname{Re} \biggl( \frac{ ( B-1 ) w(z)- ( A-1 ) }{ ( B+1 ) w(z)- ( A+1 ) } \biggr) >k\biggl\vert \frac{ ( B-1 ) w(z)- ( A-1 ) }{ ( B+1 ) w(z)- ( A+1 ) }-1\biggr\vert \biggr\} $$

or equivalently

$$\begin{aligned} \Omega_{k} [ A,B ] &= \bigl\{ u+iv: \bigl[ \bigl( B^{2}-1 \bigr) \bigl( u^{2}+v^{2} \bigr) -2 ( AB-1 ) u+ \bigl( A^{2}-1 \bigr) \bigr] ^{2} \\ &\quad >k^{2} \bigl[ \bigl( -2 ( B+1 ) \bigl( u^{2}+v^{2} \bigr) +2 ( A+B+2 ) u-2 ( A+1 ) \bigr) ^{2} \\ &\quad +4 ( A-B ) ^{2}v^{2} \bigr] \bigr\} . \end{aligned}$$

The domain $\Omega_{k} [ A,B ] $ retains the conic domain $\Omega_{k}$ inside the circular region defined by $\Omega_{0} [ A,B ] =\Omega [ A,B ] $. The impact of $\Omega [ A,B ] $ on the conic domain $\Omega_{k}$ changes the original shape of the conic regions. The ends of hyperbola and parabola get closer to each other but never meet anywhere and the ellipse gets the shape of oval. When $A\longrightarrow1$, $B\longrightarrow-1$, the radius of the circular disk defined by $\Omega [ A,B ] $ tends to infinity; consequently, the arms of hyperbola and parabola expand and the oval turns into ellipse.

### Definition 1.2

([1])

A function $f\in \mathcal{A}$ is said to be in the class $k-\mathcal{CV} [ C,D ] $, $-1\leq D< C\leq1$, if it satisfies the condition

$$ \operatorname{Re} \biggl( \frac{ ( D-1 ) \frac{ ( zf^{\prime} ( z ) ) ^{\prime}}{f^{\prime} ( z ) }- ( C-1 ) }{ ( D+1 ) \frac{ ( zf^{\prime} ( z ) ) ^{\prime}}{f^{\prime} ( z ) }- ( C+1 ) } \biggr) >k\biggl\vert \frac{ ( D-1 ) \frac{ ( zf^{\prime } ( z ) ) ^{\prime}}{f^{\prime} ( z ) }- ( C-1 ) }{ ( D+1 ) \frac{ ( zf^{\prime} ( z ) ) ^{\prime}}{f^{\prime} ( z ) }- ( C+1 ) }-1 \biggr\vert \quad ( k\geq0; z \in E ) , $$

equivalently, we can write

$$ \frac{ ( zf^{\prime} ( z ) ) ^{\prime}}{f^{ \prime} ( z ) }\in k-\mathcal{P} [ C,D ] . $$

### Definition 1.3

([1])

The class $k- \mathcal{ST} [ C,D ] $, $-1\leq D< C\leq1$, is the family of all those functions $f\in\mathcal{A}$ such that

$$ \operatorname{Re} \biggl( \frac{ ( D-1 ) \frac{zf^{\prime} ( z ) }{f ( z ) }- ( C-1 ) }{ ( D+1 ) \frac{zf^{ \prime} ( z ) }{f ( z ) }- ( C+1 ) } \biggr) >k\biggl\vert \frac{ ( D-1 ) \frac{zf^{\prime} ( z ) }{f ( z ) }- ( C-1 ) }{ ( D+1 ) \frac{zf ^{\prime} ( z ) }{f ( z ) }- ( C+1 ) }-1 \biggr\vert \quad ( k\geq0; z\in E ) , $$

or equivalently

$$ \frac{zf^{\prime} ( z ) }{f ( z ) }\in k- \mathcal{P} [ C,D ] . $$

These two classes were recently introduced by Noor and Malik [1].

Motivated by the recent work presented by Noor and Malik [1], we define some classes of analytic functions associated with conic domains as follows.

### Definition 1.4

Let $f\in\mathcal{A}$. Then $f\in k-\mathcal{UK} [ A,B,C,D ] $ if and only if there exists $g\in k-\mathcal{ST} [ C,D ] $ such that

$$ \operatorname{Re} \biggl( \frac{ ( B-1 ) \frac{zf^{\prime} ( z ) }{g ( z ) }- ( A-1 ) }{ ( B+1 ) \frac{zf^{ \prime} ( z ) }{g ( z ) }- ( A+1 ) } \biggr) >k\biggl\vert \frac{ ( B-1 ) \frac{zf^{\prime} ( z ) }{g ( z ) }- ( A-1 ) }{ ( B+1 ) \frac{zf ^{\prime} ( z ) }{g ( z ) }- ( A+1 ) }-1 \biggr\vert \quad ( k\geq0 ) , $$

or equivalently

$$ \frac{zf^{\prime} ( z ) }{g ( z ) }\in k- \mathcal{P} [ A,B ] , $$

where $-1\leq D\leq C\leq1$ and $-1\leq B< A\leq1$.

### Definition 1.5

Let $f\in\mathcal{A}$. Then $f\in k-\mathcal{UQ} [ A,B,C,D ] $ if and only if, for $-1\leq D< C\leq1$, $-1\leq B< A\leq1$ and $k \geq0$, there exists $g\in k-\mathcal{CV} [ C,D ] $ such that

$$ \operatorname{Re} \biggl( \frac{ ( B-1 ) \frac{ ( zf^{\prime} ( z ) ) ^{\prime}}{g^{\prime} ( z ) }- ( A-1 ) }{ ( B+1 ) \frac{ ( zf^{\prime} ( z ) ) ^{\prime}}{g^{\prime} ( z ) }- ( A+1 ) } \biggr) >k\biggl\vert \frac{ ( B-1 ) \frac{ ( zf^{\prime } ( z ) ) ^{\prime}}{g^{\prime} ( z ) }- ( A-1 ) }{ ( B+1 ) \frac{ ( zf^{\prime} ( z ) ) ^{\prime}}{g^{\prime} ( z ) }- ( A+1 ) }-1 \biggr\vert , $$

or equivalently

$$ \frac{ ( zf^{\prime} ( z ) ) ^{\prime}}{g^{ \prime} ( z ) }\in k-\mathcal{P} [ A,B ] . $$

It can easily be seen that

1.5 $$ f\in k\mathcal{-UQ} [ A,B,C,D ] \Leftrightarrow zf^{\prime} \in k \mathcal{-UK} [ A,B,C,D ] . $$

*Special cases:*

i. $0-\mathcal{UK} [ A,B,C,D ] =\mathcal{K} [ A,B,C,D ] $ and $0-\mathcal{UQ} [ A,B,1,-1 ] = \mathcal{Q} [ A,B ] $, subclasses of close-to-convex and quasi-convex functions studied by Silvia and Noor, respectively, see [15, 16].
ii. $k-\mathcal{UK} [ 1,-1,1,-1 ] =k-\mathcal{UK}$ and $k-\mathcal{UQ} [ 1,-1,1,-1 ] =k-\mathcal{UQ}$, the class of k-uniformly close-to-convex and the class of k-uniformly quasi-convex functions studied by Acu [17].
iii. $k-\mathcal{UK} [ 1-2\beta,-1,1-2\gamma,-1 ] =k- \mathcal{UK} ( \beta,\gamma ) $ and $k-\mathcal{UQ} [ 1-2 \beta,-1,1-2\gamma,-1 ] =k-\mathcal{UQ} ( \beta,\gamma ) $, the well-known class of k-uniformly close-to-convex and the class of k-uniformly quasi-convex functions of order *β* and type *γ*, see [18].
iv. $0\mathcal{-UK} [ 1-2\beta,-1,1-2\gamma,-1 ] = \mathcal{K} ( \beta,\gamma ) $ and $0-\mathcal{UQ} [ 1-2 \beta,-1,1-2\gamma,-1 ] =\mathcal{Q} ( \beta,\gamma ) $, well-known classes of close-to-convex and quasi-convex functions of order *β* type *γ*, see [19, 20].
v. $0-\mathcal{UK} [ 1,-1,1,-1 ] =\mathcal{K}$ and $0-\mathcal{UQ} [ 1,-1,1,-1 ] =\mathcal{Q}$, the classes of close-to-convex and quasi-convex functions; for details, see [21, 22].

Throughout this paper, we assume that $-1\leq D< C\leq1$, $-1\leq B< A \leq1$ and $k\geq0$ unless otherwise specified.

## A set of lemmas

To prove our main results, we need the following lemmas.

### Lemma 2.1

([23])

*Let* $p ( z ) =1+\sum_{n=1}^{\infty}p_{n}z^{n}\prec F ( z ) =1+\sum_{n=1}^{\infty}d_{n}z^{n}$ *in* *E*. *If* $F ( z ) $ *is univalent in* *E* *and* $F ( E ) $ *is convex*, *then*

$$ \vert p_{n}\vert \leq \vert d_{1}\vert ,\quad n\geq1. $$

### Lemma 2.2

([1])

*Let* $p ( z ) =1+\sum_{n=1}^{\infty}c_{n}z^{n}\in k- \mathcal{P} [ A,B ] $. *Then*

$$ \vert c_{n}\vert \leq\bigl\vert \delta ( A,B,k ) \bigr\vert , $$

*where*

2.1 $$ \delta ( A,B,k ) =\frac{ ( A-B ) \delta_{k}}{2}, $$

*and*

2.2 $$ \delta_{k}= \textstyle\begin{cases} \frac{8 ( \cos^{-1}k ) ^{2}}{\pi^{2} ( 1-k^{2} ) }, &0\leq k< 1, \\ \frac{8}{\pi^{2}}, &k=1, \\ \frac{\pi^{2}}{4\sqrt{t} ( k^{2}-1 ) R^{2} ( t ) ( 1+t ) }, &k>1. \end{cases} $$

### Lemma 2.3

([2])

*Let* *f* *and* *g* *be in the class* $\mathcal{C}$ *and* $\mathcal{S}^{ \ast}$, *respectively*. *Then*, *for every function* $F ( z ) $ *analytic in* *E* *with* $F ( 0 ) =1$, *we have*

$$ \frac{f ( z ) \ast g ( z ) F ( z ) }{f ( z ) \ast g ( z ) }\in\overline{\operatorname{co}} \bigl( F ( E ) \bigr) , \quad z\in E, $$

*where “*∗*” denotes the well*-*known convolution of two analytic functions and* $\overline{\operatorname{co}}F ( E ) $ *denotes the closed convex hull* $F ( E ) $.

### Lemma 2.4

([1])

*Let* $g\in k\mathcal{-ST} [ C,D ] $ *with* $k\geq0 $ *and be given by*

$$ g ( z ) =z+\sum_{n=2}^{\infty}b_{n}z^{n}. $$

*Then*

$$ \vert b_{n}\vert \leq\prod_{j=0}^{n-2} \frac{\vert ( C-D ) \delta_{k}-2jD\vert }{2 ( j+1 ) }, $$

*where* $\delta_{k}$ *is defined by* (2.2).

## The main results and their consequences

This section is about the main results of our defined families $k\mathcal{-UK} [ A,B,C,D ] $ and $k-\mathcal{UQ} [ A,B,C,D ] $. These families will be thoroughly investigated by studying their important properties including coefficient inequalities, sufficient condition, necessary condition, arc length problem, the growth rate of coefficients, convolution preserving properties and the radius of convexity problem. We will also discuss some special cases of our main results.

1. *Coefficient inequalities*

### Theorem 3.1

*Let* $f\in k\mathcal{-UK} [ A,B,C,D ] $, *and let it be of the form given by* (1.1). *Then*, *for* $n\geq2$, *we have*

$$ \vert a_{n}\vert \leq\frac{1}{n}\prod _{i=0}^{n-2}\frac{ \vert ( C-D ) \delta_{k}-2iD\vert }{2 ( i+1 ) }+ \frac{ ( A-B ) \vert \delta_{k}\vert }{2n} \sum _{j=1}^{n-1}\prod _{i=0}^{j-2}\frac{\vert ( C-D ) \delta_{k}-2iD\vert }{2 ( i+1 ) }, $$

*where* $\delta_{k}$ *is defined by* (2.2). *This result is not sharp*.

### Proof

Let us take

3.1 $$ zf^{\prime} ( z ) =g ( z ) p ( z ) , $$

where $p\in k-\mathcal{P} [ A,B ] $ and $g\in k-\mathcal{ST} [ C,D ] $. Let $zf^{\prime} ( z ) =z+\sum_{n=2}^{\infty}na_{n}z^{n}$, $g ( z ) =z+\sum_{n=2} ^{\infty}b_{n}z^{n}$ and $p ( z ) =1+\sum_{n=1}^{ \infty}c_{n}z^{n}$. Then (3.1) becomes

$$ z+\sum_{n=2}^{\infty}na_{n}z^{n}= \Biggl( z+\sum_{n=2} ^{\infty}b_{n}z^{n} \Biggr) \Biggl( 1+\sum_{n=1}^{\infty}c_{n}z ^{n} \Biggr) . $$

Equating the coefficients of $z^{n}$ on both sides, we have

$$ na_{n}=b_{n}+\sum_{j=1}^{n-1}b_{j}c_{n-j}. $$

This implies that

3.2 $$ n\vert a_{n}\vert \leq \vert b_{n}\vert + \sum _{j=1}^{n-1}\vert b_{j}\vert \vert c_{n-j}\vert . $$

Since $p\in k-\mathcal{P} [ A,B ] $ and $g\in k-\mathcal{ST} [ C,D ] $, therefore by Lemma 2.2 and Lemma 2.4 we have

$$ \vert b_{n}\vert \leq\prod_{i=0}^{n-2} \frac{\vert ( C-D ) \delta_{k}-2iD\vert }{2 ( i+1 ) } $$

and

$$ \vert c_{n}\vert \leq\frac{1}{2} ( A-B ) \vert \delta _{k}\vert . $$

Hence (3.2) becomes

$$ n\vert a_{n}\vert \leq\prod_{i=0}^{n-2} \frac{\vert ( C-D ) \delta_{k}-2iD\vert }{2 ( i+1 ) }+\sum_{j=1}^{n-1} \frac{ ( A-B ) \vert \delta_{k}\vert }{2} \prod_{i=0}^{j-2} \frac{\vert ( C-D ) \delta_{k}-2iD\vert }{2 ( i+1 ) }, $$

which implies that

$$ \vert a_{n}\vert \leq\frac{1}{n}\prod _{i=0}^{n-2}\frac{ \vert ( C-D ) \delta_{k}-2iD\vert }{2 ( i+1 ) }+ \frac{ ( A-B ) \vert \delta_{k}\vert }{2n} \sum _{j=1}^{n-1}\prod _{i=0}^{j-2}\frac{\vert ( C-D ) \delta_{k}-2iD\vert }{2 ( i+1 ) }. $$

This completes the proof. □

### Corollary 3.2

([9])

*Let* $f\in k-\mathcal{UK} [ 1,-1,1,-1 ] $ *which has the form* (1.1). *Then*, *for* $n\geq2$,

$$ \vert a_{n}\vert \leq\frac{ ( \vert \delta_{k}\vert ) _{n-1}}{n!}+\frac{\vert \delta _{k}\vert }{n}\sum _{j=0}^{n-1}\frac{ ( \vert \delta _{k}\vert ) _{j-1}}{ ( j-1 ) !}. $$

### Corollary 3.3

*Let* $f\in k-\mathcal{UK} [ 1-2\beta,-1,1-2\gamma,-1 ] =f \in k-\mathcal{UK} [ \beta,\gamma ] $ *which has the form* (1.1). *Then*, *for* $n\geq2$,

$$ \vert a_{n}\vert \leq\frac{ ( \vert \delta_{k}\vert ) _{n-1}}{n!}+\frac{\vert \delta _{k}\vert }{n}\sum _{j=0}^{n-1}\frac{ ( \vert \delta _{k}\vert ) _{j-1}}{ ( j-1 ) !}. $$

### Corollary 3.4

([21])

*Let* $f\in0-\mathcal{UK} [ 1,-1,1,-1 ] = \mathcal{K}$ *which has the form* (1.1). *Then*, *for* $n\geq2$,

$$ \vert a_{n}\vert \leq n. $$

Using relation (1.5) and Theorem 3.1, we obtain immediately the following result.

### Theorem 3.5

*Let* $f\in k-\mathcal{UQ} [ A,B,C,D ] $ *which has the form* (1.1). *Then*, *for* $n\geq2$,

$$ \vert a_{n}\vert \leq\frac{1}{n^{2}}\prod _{i=0} ^{n-2}\frac{\vert ( C-D ) \delta_{k}-2iD\vert }{2 ( i+1 ) }+\frac{ ( A-B ) \vert \delta_{k}\vert }{2n ^{2}}\sum _{j=1}^{n-1}\prod _{i=0}^{j-2}\frac{\vert ( C-D ) \delta_{k}-2iD\vert }{2 ( i+1 ) }. $$

By assigning different permissible values to the parameters, we obtain several known results, see [17, 21, 22].

2. *Sufficient conditions*

### Theorem 3.6

*Let* $f\in\mathcal{A}$ *and be given by* (1.1). *Then* $f\in k\mathcal{-UK} [ A,B,C,D ] $ *if*

3.3 $$ \sum_{n=2}^{\infty} \bigl[ 2 ( k+1 ) \vert b_{n}-na _{n}\vert +\bigl\vert ( B+1 ) na_{n}- ( A+1 ) b_{n}\bigr\vert \bigr] < \vert B-A\vert . $$

### Proof

Let us assume that equation (3.3) holds true. It is sufficient to show that

$$ k\biggl\vert \frac{ ( B-1 ) \frac{zf^{\prime} ( z ) }{g ( z ) }- ( A-1 ) }{ ( B+1 ) \frac{zf ^{\prime} ( z ) }{g ( z ) }- ( A+1 ) }-1\biggr\vert - \operatorname{Re} \biggl( \frac{ ( B-1 ) \frac{zf^{\prime} ( z ) }{g ( z ) }- ( A-1 ) }{ ( B+1 ) \frac{zf^{ \prime} ( z ) }{g ( z ) }- ( A+1 ) }-1 \biggr) < 1. $$

Now consider

$$\begin{aligned} \biggl\vert \frac{ ( B-1 ) \frac{zf^{\prime} ( z ) }{g ( z ) }- ( A-1 ) }{ ( B+1 ) \frac{zf^{ \prime} ( z ) }{g ( z ) }- ( A+1 ) }-1\biggr\vert =& \biggl\vert \frac{ ( B-1 ) zf^{\prime} ( z ) - ( A-1 ) g ( z ) }{ ( B+1 ) zf^{\prime} ( z ) - ( A+1 ) g ( z ) }-1\biggr\vert \\ =&2\biggl\vert \frac{g ( z ) -zf^{\prime} ( z ) }{ ( B+1 ) zf^{\prime} ( z ) - ( A+1 ) g ( z ) }\biggr\vert \\ =&2\biggl\vert \frac{\sum_{n=2}^{\infty} ( b_{n}-na_{n} ) z^{n}}{ ( B-A ) z+\sum_{n=2}^{ \infty} [ ( B+1 ) na_{n}- ( A+1 ) b_{n} ] z^{n}}\biggr\vert \\ \leq&\frac{2\sum_{n=2}^{\infty} \vert b_{n}-na_{n}\vert }{ ( B-A ) -\sum_{n=2}^{\infty} \vert ( B+1 ) na_{n}- ( A+1 ) b_{n}\vert }. \end{aligned}$$

Since

$$\begin{aligned}& k\biggl\vert \frac{ ( B-1 ) \frac{zf^{\prime} ( z ) }{g ( z ) }- ( A-1 ) }{ ( B+1 ) \frac{zf ^{\prime} ( z ) }{g ( z ) }- ( A+1 ) }-1\biggr\vert - \operatorname{Re} \biggl( \frac{ ( B-1 ) \frac{zf^{\prime} ( z ) }{g ( z ) }- ( A-1 ) }{ ( B+1 ) \frac{zf^{ \prime} ( z ) }{g ( z ) }- ( A+1 ) }-1 \biggr) \\& \quad \leq ( k+1 ) \biggl\vert \frac{ ( B-1 ) \frac{zf ^{\prime} ( z ) }{g ( z ) }- ( A-1 ) }{ ( B+1 ) \frac{zf^{\prime} ( z ) }{g ( z ) }- ( A+1 ) }-1\biggr\vert \leq \frac{2 ( k+1 ) \sum_{n=2}^{\infty} \vert b_{n}-na_{n}\vert }{ ( B-A ) -\sum_{n=2}^{\infty} \vert ( B+1 ) na _{n}- ( A+1 ) b_{n}\vert }. \end{aligned}$$

The last inequality is bounded by 1 if

$$ 2 ( k+1 ) \sum_{n=2}^{\infty} \vert b_{n}-na_{n}\vert \leq \vert B-A\vert -\sum _{n=2}^{\infty}\bigl\vert ( B+1 ) na_{n}- ( A+1 ) b_{n}\bigr\vert . $$

Hence we have

$$ \sum_{n=2}^{\infty} \bigl[ 2 ( k+1 ) \vert b_{n}-na _{n}\vert +\bigl\vert ( B+1 ) na_{n}- ( A+1 ) b_{n}\bigr\vert \bigr] \leq \vert B-A\vert . $$

This completes the proof. □

### Theorem 3.7

*Let* $f\in\mathcal{A}$ *which has the form* (1.1). *Then* $f\in k-\mathcal{UQ} [ A,B,C,D ] $ *if*

$$ \sum_{n=2}^{\infty}n \bigl[ 2 ( k+1 ) \vert b _{n}-na_{n}\vert +\bigl\vert ( B+1 ) na_{n}- ( A+1 ) b_{n}\bigr\vert \bigr] < \vert B-A\vert . $$

The proof follows immediately by using Theorem 3.6 and relation (1.5).

### Corollary 3.8

([24])

*A function is said to be in the class* $1- \mathcal{UQ} [ 1-2\beta,-1,1,-1 ] =\mathcal{UQ} ( \beta ) $ *for* $g ( z ) =z$ *if*

$$ \sum_{n=2}^{\infty}n^{2}\vert a_{n}\vert < \frac{1- \beta}{2}. $$

3. *Necessary condition*

### Theorem 3.9

*Let* $f\in k-\mathcal{UK} [ A,B,C,D ] $. *Then*, *for* $\theta_{1}< \theta_{2}$, $z\in E$,

$$ \int_{\theta_{1}}^{\theta_{2}}\operatorname{Re} \biggl\{ \frac{ ( zf^{ \prime} ( z ) ) ^{\prime}}{f^{\prime} ( z ) } \biggr\} \,d\theta>- \biggl[ \frac{C-D}{2k+1-D}+\lambda \biggr] \pi, $$

*where* *λ* *is defined by* (1.4).

### Proof

Since $f\in k-\mathcal{UK} [ A,B,C,D ] $, there exists $g\in k-\mathcal{CV} [ C,D ] \subset\mathcal{C} ( \beta _{1} ) $,

3.4 $$ \beta_{1}=\frac{2k+1-C}{2k+1-D} $$

such that

$$ f^{\prime} ( z ) =g^{\prime} ( z ) p ( z ) , $$

where $p\in k-\mathcal{P} [ A,B ] $. We can write

$$ f^{\prime} ( z ) = \bigl( g_{1}^{\prime} ( z ) \bigr) ^{1-\beta_{1}}h^{\lambda} ( z ) , \quad h\in \mathcal{P} [ A,B ] \subset \mathcal{P}, g_{1}\in \mathcal{C}. $$

For $z=re^{i\theta}$, $0\leq r<1$, $0\leq\theta_{1}\leq\theta_{2} \leq2\pi$, we have

3.5 $$ \begin{aligned}[b] \int_{\theta_{1}}^{\theta_{2}}\operatorname{Re} \biggl\{ \frac{ ( zf^{ \prime} ( z ) ) ^{\prime}}{f^{\prime} ( z ) } \biggr\} \,d\theta&= ( 1-\beta_{1} ) \int_{\theta_{1}} ^{\theta_{2}}\operatorname{Re} \biggl\{ \frac{ ( zg_{1}^{\prime} ( z ) ) ^{\prime}}{g_{1}^{\prime} ( z ) } \biggr\} \,d\theta +\beta_{1} ( \theta_{2}- \theta_{1} ) \\ &\quad{} +\lambda \int _{\theta_{1}}^{\theta_{2}}\operatorname{Re} \biggl\{ \frac{zh^{\prime} ( z ) }{h ( z ) } \biggr\} \,d\theta. \end{aligned} $$

Also, we observe that, for $h\in\mathcal{P} [ A,B ] $,

$$\begin{aligned} \frac{\partial}{\partial\theta}\arg h \bigl( re^{i\theta} \bigr) =& \frac{ \partial}{\partial\theta}\operatorname{Re} \bigl\{ -i\ln h \bigl( re^{i\theta} \bigr) \bigr\} \\ =&\operatorname{Re} \biggl\{ \frac{re^{i\theta}h^{\prime} ( re^{i\theta} ) }{h ( re^{i\theta} ) } \biggr\} . \end{aligned}$$

Therefore

$$ \int_{\theta_{1}}^{\theta_{2}}\operatorname{Re} \biggl\{ \frac{re^{i\theta}h ^{\prime} ( re^{i\theta} ) }{h ( re^{i\theta} ) } \biggr\} \,d\theta=\arg h \bigl( re^{i\theta_{2}} \bigr) -\arg h \bigl( re^{i\theta_{1}} \bigr) , $$

this implies that

3.6 $$ \max_{h\in P [ A,B ] }\biggl\vert \int_{\theta_{1}} ^{\theta_{2}}\operatorname{Re} \biggl\{ \frac{re^{i\theta}h^{\prime} ( re^{i \theta} ) }{h ( re^{i\theta} ) } \biggr\} \,d\theta \biggr\vert = \max_{h\in P [ A,B ] }\bigl\vert \arg h \bigl( re^{i\theta_{2}} \bigr) -\arg h \bigl( re^{i\theta_{1}} \bigr) \bigr\vert . $$

Since $h\in\mathcal{P} [ A,B ] $, so

$$ \biggl\vert h ( z ) -\frac{1-ABr^{2}}{1-B^{2}r^{2}}\biggr\vert \leq\frac{ ( A-B ) r}{1-B^{2}r^{2}}. $$

From (3.6), we observe that

3.7 $$\begin{aligned} \max_{h\in P [ A,B ] }\biggl\vert \int_{\theta_{1}} ^{\theta_{2}}\operatorname{Re} \biggl\{ \frac{re^{i\theta}h^{\prime} ( re^{i \theta} ) }{h ( re^{i\theta} ) } \biggr\} \,d\theta \biggr\vert \leq&2\sin^{-1} \biggl( \frac{ ( A-B ) r}{1-ABr^{2}} \biggr) \\ \leq&\pi-2\cos^{-1} \biggl( \frac{ ( A-B ) r}{1-ABr^{2}} \biggr) . \end{aligned}$$

Also, for $g_{1}\in\mathcal{C}$, we have

$$ \int_{\theta_{1}}^{\theta_{2}}\operatorname{Re} \biggl\{ \frac{ ( zg_{1} ^{\prime} ( z ) ) ^{\prime}}{g_{1}^{\prime} ( z ) } \biggr\} \,d\theta>-\pi. $$

Using (3.6) and (3.7) in (3.5), we obtain

$$\begin{aligned} \int_{\theta_{1}}^{\theta_{2}}\operatorname{Re} \biggl\{ \frac{ ( zf^{ \prime} ( z ) ) ^{\prime}}{f^{\prime} ( z ) } \biggr\} \,d\theta >&- ( 1-\beta_{1} ) \pi+\beta_{1} ( \theta_{2}-\theta_{1} ) -\lambda\pi+2\lambda \cos^{-1} \biggl( \frac{ ( A-B ) r}{1-ABr^{2}} \biggr) \\ >&- \biggl[ \frac{ ( C-D ) }{2k+1-D}+\lambda \biggr] \pi ( r\rightarrow1 ) . \end{aligned}$$

This completes the required result. □

### Remark 3.10

Let $f\in k-\mathcal{UK} [ A,B,C,D ] $. Then, for $\frac{C-D}{2k+1-D}<1-\lambda$,

$$ \int_{\theta_{1}}^{\theta_{2}}\operatorname{Re} \biggl\{ \frac{ ( zf^{ \prime} ( z ) ) ^{\prime}}{f^{\prime} ( z ) } \biggr\} \,d\theta>-\pi, $$

and hence *f* is univalent in *E*, see [21].

### Corollary 3.11

([21])

*Let* $f\in0-\mathcal{UK} [ 1,-1,1,-1 ] $. *Then*, *for* $\theta_{1}<\theta_{2}$, $z\in E$,

$$ \int_{\theta_{1}}^{\theta_{2}}\operatorname{Re} \biggl\{ \frac{ ( zf^{ \prime} ( z ) ) ^{\prime}}{f^{\prime} ( z ) } \biggr\} \,d\theta>-\pi. $$

4. *Arc length problem*

### Theorem 3.12

*Let* $f\in k-\mathcal{UK} [ A,B,C,D ] $ *which has the form* (1.1). *Then*

$$ \mathcal{L}_{r} ( f ) \leq C ( \lambda,C,D ) \biggl( \frac{1}{1-r} \biggr) ^{2 ( 1-\beta_{1} ) -1}\quad ( r\rightarrow1 ) , $$

*where* $\beta_{1}$ *is defined by* (3.4) *and* $C ( \lambda,A,B ) $ *is a constant depending upon* *λ*, *A* *and B*.

### Proof

Let

3.8 $$ zf^{\prime} ( z ) =g ( z ) h^{\lambda} ( z ) , $$

where $g\in k-\mathcal{ST} [ C,D ] $ and $h\in\mathcal{P} [ A,B ] \subset\mathcal{P}$. Since $k-\mathcal{ST} [ C,D ] \subseteq\mathcal{S}^{\ast} ( \beta_{1} ) $, see [1]. We can write

$$ g ( z ) =z \biggl( \frac{g_{1} ( z ) }{z} \biggr) ^{1- \beta_{1}},\quad g_{1} \in\mathcal{S}^{\ast}. $$

Equation (3.8) gives

$$ zf^{\prime} ( z ) =z^{\beta_{1}} \bigl( g_{1} ( z ) \bigr) ^{1-\beta_{1}}h^{\lambda} ( z ) . $$

Now, for $z=re^{i\theta}$,

$$ \mathcal{L}_{r} ( f ) = \int_{0}^{2\pi}\bigl\vert zf ^{\prime} ( z ) \bigr\vert \,d\theta= \int_{0}^{2 \pi}\bigl\vert z^{\beta_{1}} \bigl( g_{1} ( z ) \bigr) ^{1- \beta_{1}}h^{\lambda} ( z ) \bigr\vert \,d\theta. $$

Using Holder’s inequality, we have

3.9 $$ \mathcal{L}_{r} ( f ) \leq2\pi \biggl( \frac{1}{2\pi} \int_{0}^{2\pi}\bigl\vert \bigl( g_{1} ( z ) \bigr) ^{ ( 1-\beta_{1} ) ( \frac{2}{2-\lambda} ) }\bigr\vert \,d\theta \biggr) ^{\frac{2-\lambda}{2}} \biggl( \frac{1}{2\pi} \int _{0}^{2\pi}\bigl\vert h ( z ) \bigr\vert ^{2}\,d\theta \biggr) ^{\frac{\lambda}{2}}. $$

Since $h\in\mathcal{P} [ A,B ] \subset\mathcal{P}$, so

3.10 $$ \frac{1}{2\pi} \int_{0}^{2\pi}\bigl\vert h ( z ) \bigr\vert ^{2}\,d\theta\leq\frac{1+ \{ ( A-B ) ^{2}-1 \} r ^{2}}{1-r^{2}}. $$

Using (3.10) and the distortion result for a starlike function in (3.9), we obtain

$$\begin{aligned} \mathcal{L}_{r} ( f ) \leq&2\pi \biggl( \frac{1}{2\pi} \int_{0}^{2\pi}\frac{r^{ ( 1-\beta_{1} ) ( \frac{2}{2- \lambda} ) }}{\vert 1-re^{i\theta} \vert ^{\frac{4 ( 1-\beta_{1} ) }{2-\lambda}}}\,d\theta \biggr) ^{\frac{2- \lambda}{2}} \biggl( \frac{1+ \{ ( A-B ) ^{2}-1 \} r ^{2}}{1-r^{2}} \biggr) ^{\frac{\lambda}{2}} \\ \leq&C ( \lambda,A,B ) \biggl( \frac{1}{1-r} \biggr) ^{2 ( 1-\beta_{1} ) +\lambda-1} \quad ( r \rightarrow1 ) , \end{aligned}$$

where $C ( \lambda,A,B ) =\pi^{\frac{\lambda}{2}} ( A-B ) ^{\lambda}$ and $2 ( 1-\beta_{1} ) +\lambda>1$. This completes the proof. □

### Corollary 3.13

([25])

*Let* $f\in0-\mathcal{UK} [ 1,-1,1,-1 ] $. *Then*, *for* $0< r<1$,

$$ \mathcal{L}_{r} ( f ) \leq\mathcal{O} \biggl( \mathcal{M} ( r ) \log \frac{1}{1-r} \biggr) \quad \textit{as }r\rightarrow1, $$

*where* $\mathcal{O}$-*notation denotes that the constant is absolute and* $\mathcal{M} ( r ) =\max_{\vert z\vert =r}\vert f ( z ) \vert $.

5. *Growth rate of coefficients*

### Theorem 3.14

*Let* $f\in k-\mathcal{UK} [ A,B,C,D ] $ *which has the form* (1.1). *Then*, *for* $k\geq0$, *we have*

$$ \vert a_{n}\vert \leq C ( \lambda,A,B ) n^{2 ( 1-\beta_{1} ) +\lambda-2}, $$

*where* $\beta_{1}$ *is defined by* (3.4).

### Proof

From Cauchy’s theorem with $z=re^{i\theta}$, one can easily have

$$ na_{n}=\frac{1}{2\pi r^{n}} \int_{0}^{2\pi}zf^{\prime} ( z ) e^{-in\theta} \,d\theta. $$

This implies that

$$\begin{aligned} \vert na_{n}\vert \leq&\frac{1}{2\pi r^{n}} \int _{0}^{2\pi}\bigl\vert zf^{\prime} ( z ) \bigr\vert \, d\theta \\ =&\frac{1}{2\pi r^{n}}\mathcal{L}_{r} ( f ) . \end{aligned}$$

Using Theorem 3.12 and putting $r=1-\frac{1}{n}$, we obtain the required result. □

6. *Convolution properties*

### Theorem 3.15

*If* $f\in k-\mathcal{ST} [ C,D ] $ *and* $\varphi\in \mathcal{C}$, *then* $\varphi\ast f\in k-\mathcal{ST} [ C,D ] $.

### Proof

To prove the result, we need to prove

$$ \frac{z ( \varphi ( z ) \ast f ( z ) ) ^{\prime}}{\varphi ( z ) \ast f ( z ) }\in k- \mathcal{P} [ A,B ] . $$

Consider

$$\begin{aligned} \frac{z ( \varphi ( z ) \ast f ( z ) ) ^{\prime}}{\varphi ( z ) \ast f ( z ) } =&\frac{ \varphi ( z ) \ast\frac{zf^{\prime} ( z ) }{f ( z ) }f ( z ) }{\varphi ( z ) \ast f ( z ) } \\ =&\frac{\varphi ( z ) \ast\Psi ( z ) f ( z ) }{\varphi ( z ) \ast f ( z ) }, \end{aligned}$$

where $\frac{zf^{\prime} ( z ) }{f ( z ) }=\Psi ( z ) \in k-\mathcal{P} [ C,D ] $. Applying Lemma 2.3, we obtain the required result. □

### Theorem 3.16

*Let* $f\in k-\mathcal{UK} [ A,B,C,D ] $ *and* $\varphi\in \mathcal{C}$. *Then* $\varphi\ast f\in k-\mathcal{UK} [ A,B,C,D ] $.

### Proof

Since $f\in k-\mathcal{UK} [ A,B,C,D ] $, there exists $g\in k-\mathcal{ST} [ C,D ] $ such that $\frac{zf^{\prime} ( z ) }{g ( z ) }\in k-\mathcal{P} [ A,B ] $. It follows from Lemma 2.3 that $\varphi\ast g\in k- \mathcal{ST} [ C,D ] $. Now

$$\begin{aligned} \frac{z ( \varphi ( z ) \ast f ( z ) ) ^{\prime}}{\varphi ( z ) \ast g ( z ) } =&\frac{ \varphi ( z ) \ast zf^{\prime} ( z ) }{\varphi ( z ) \ast g ( z ) }=\frac{\varphi ( z ) \ast\frac{zf^{\prime} ( z ) }{g ( z ) }g ( z ) }{\varphi ( z ) \ast g ( z ) } \\ =&\frac{\varphi ( z ) \ast F ( z ) g ( z ) }{\varphi ( z ) \ast g ( z ) }, \end{aligned}$$

where $F ( z ) \in k-\mathcal{P} [ A,B ] $. Applying Lemma 2.3, we have $\frac{z ( \varphi ( z ) \ast f ( z ) ) ^{\prime}}{\varphi ( z ) \ast g ( z ) }\in k-\mathcal{UK} [ A,B,C,D ] $ for $z\in E$. □

7. *Radius of convexity problem*

### Theorem 3.17

*Let* $f\in k-\mathcal{UK} [ A,B,C,D ] $ *in* *E*. *Then* $f\in \mathcal{C}$ *for* $\vert z\vert < r_{1}$, *where*

3.11 $$ r_{1}=\frac{2}{2 ( 1-\beta_{1} ) +\lambda ( A-B ) +\sqrt{ ( [ 2 ( 1-\beta_{1} ) +\lambda ( A-B ) ] ^{2}+4 [ 2\beta_{1}-1 ] ) }}, $$

*where* *λ* *and* $\beta_{1}$ *are defined by* (1.4) *and* (3.4), *respectively*.

### Proof

Let

$$ zf^{\prime} ( z ) =g ( z ) p ( z ) , $$

where $g\in k-\mathcal{ST} [ C,D ] $ and $p\in k-\mathcal{P} [ A,B ] $. Since $k-\mathcal{ST} [ C,D ] \subset \mathcal{S}^{\ast} ( \beta_{1} ) $, it is known [26] that there exists $g_{1}\in\mathcal{S}^{\ast}$ such that

$$ g ( z ) =z \biggl( \frac{g_{1} ( z ) }{z} \biggr) ^{1- \beta_{1}}. $$

We can write

3.12 $$ zf^{\prime} ( z ) =z^{\beta_{1}} \bigl( g_{1} ( z ) \bigr) ^{1-\beta_{1}}h^{\lambda} ( z ) , \quad h \in\mathcal{P} [ A,B ] \subset \mathcal{P}. $$

The logarithmic differentiation of (3.12) yields

$$ \frac{ ( zf^{\prime} ( z ) ) ^{\prime}}{f^{ \prime} ( z ) }=\beta_{1}+ ( 1-\beta_{1} ) \frac{zg _{1}^{\prime} ( z ) }{g_{1} ( z ) }+\lambda\frac{zh ^{\prime} ( z ) }{h ( z ) }. $$

Using the distortion result for the classes $\mathcal{S}^{\ast}$ and $\mathcal{P} [ A,B ] $, we obtain

3.13 $$\begin{aligned} \operatorname{Re}\frac{ ( zf^{\prime} ( z ) ) ^{\prime}}{f ^{\prime} ( z ) } \geq&\beta_{1}+ ( 1-\beta_{1} ) \frac{1-r}{1+r}-\frac{\lambda ( A-B ) r}{1-r^{2}} \\ \geq&\frac{1+ ( 1-2\beta_{1} ) r^{2}- [ 2 ( 1-\beta_{1} ) +\lambda ( A-B ) ] r}{1-r^{2}}. \end{aligned}$$

The right-hand side of (3.13) is positive for $\vert z\vert < r _{1}$, where $r_{1}$ is given by (3.11). □

We note the following cases:

(i). For $A=1$, $B=-1$, $C=1$ and $D=-1$, we obtain the radius of convexity problem for the class $k-\mathcal{UK}$.
(ii). For $k=0$, we have the radius of convexity for the class $\mathcal{K} [ A,B,C,D ] $.
(iii). For $A=1$, $B=-1$, $C=1$, $D=-1$ and $k=0$, we have the radius of convexity problem for the well-known class of close-to-convex functions studied by Kaplan [21].
